# Randomized controlled trial protocol on enhancing students’ togetherness, relatedness, and interactions for learning in physical education: the TRI-PE project

**DOI:** 10.3389/fpsyg.2025.1629158

**Published:** 2025-08-26

**Authors:** Rubén Llanos-Muñoz, Javier Sevil-Serrano, David Lobo-Triviño, Miguel A. López-Gajardo, Francisco M. Leo

**Affiliations:** 1 Departmento de Didáctica de la Expresión Musical, Plástica y Corporal, Facultad de Formación del Profesorado, Universidad de Extremadura, Cáceres, Spain; 2 Departmento de Didáctica de la Expresión Musical, Plástica y Corporal, Facultad de Ciencias del Deporte, Universidad de Extremadura, Cáceres, Spain

**Keywords:** group dynamics, intervention, need-support, peer interactions, psychological needs, relatedness support

## Abstract

Grounded in Self-Determination Theory and the Conceptual Model of Cohesion, relatedness satisfaction and class cohesion have been positively linked to learning-related outcomes. However, the benefits for both teachers and students of a training program focused on improving Physical Education (PE) teachers’ relatedness-supportive behaviors and class cohesion have not yet been explored. This study presents the protocol for a training program designed to provide PE teachers with relatedness-supportive and class cohesion strategies, as well as to avoid relatedness-thwarting strategies. Consequently, this program aims to enhance teachers’ interpersonal style and students’ motivational and (mal)adaptive outcomes in PE lessons. A randomized controlled trial with a mixed-methods approach will be conducted as part of a three-wave longitudinal study. Between 8–10 secondary PE teachers and their students will be assigned to either the experimental group or the control group. The face-to-face training program implemented with experimental group’s teachers will consist of two group sessions, one group booster session, and two individual follow-up sessions. After completing the training, teachers will implement the strategies over approximately six months during their PE classes. Beliefs, feasibility, and intention to apply the strategies, relatedness-supportive behaviors, relationship satisfaction with students, class cohesion, motivational variables, and (mal)adaptive outcomes will be assessed in PE teachers and their students at three distinct time points: before the training program (Time 1), at the end of the implementation (Time 2), and 2 months later (Time 3). Additionally, a focus group involving all experimental PE teachers will be held at the end of the implementation (T2). The results of this study will help determine whether this type of training program can benefit both students and teachers.

## Introduction

A body of research emphasizes that both the teacher–student bond and peer interactions are key factors in the teaching–learning process (Leo et al., 2023d; Sparks et al., 2016, 2017; Vasconcellos et al., 2020; White et al., 2021). Consequently, Spanish educational policies emphasize the importance of working through collaborative and cooperative methodologies via peer learning (Spanish Government, 2020). To analyze these interactions, several theoretical approaches, such as Self-Determination Theory (SDT; Deci and Ryan, 1985, 2000) and the Conceptual Model of Cohesion (CMC; Carron et al., 1985), have examined student-teacher and student–student relationships in the classroom through the satisfaction/frustration of relatedness and class cohesion, respectively. Previous SDT-based interventions in Physical Education (PE) have targeted the support of autonomy, competence, and relatedness needs to promote student motivation and engagement (Vasconcellos et al., 2020). However, to our knowledge, there is only one SDT-based training program for PE teachers, focused exclusively on providing relatedness-supportive strategies to their students, but it was also not centered on improving group cohesion strategies (Sparks et al., 2017). Accordingly, this study describes the protocol of a professional development program that provides PE teachers with strategies to support relatedness and foster class cohesion.

### The role of relatedness in Self-Determination Theory

According to SDT, levels of self-determined motivation are shaped by the satisfaction or frustration of three essential, universal, and innate psychological needs—autonomy, competence, and relatedness (Deci and Ryan, 2000). Specifically, the sub-theory of Relationship Motivation Theory (RMT; Deci and Ryan, 2014) postulates the existence of a basic psychological need for relatedness, which drives individuals to seek and strengthen affective bonds. RMT asserts that “establishing and maintaining close relationships are among the most important and autonomously pursued aspects of people’s lives” (Deci and Ryan, 2014, p. 53). This need must be supported by the social environment (e.g., teachers, families, and peers) to generate positive effects in affective, behavioral, and cognitive domains (Vasconcellos et al., 2020; White et al., 2021; Leo et al., 2023b).

In the educational context, teachers play a crucial role in fostering classroom-relatedness. Specifically, PE teachers can use relatedness-supportive and/or relatedness-thwarting strategies (Ahmadi et al., 2023). In the first case, teachers who foster relatedness create a warm environment that facilitates positive communicative exchanges between students and teachers, as well as among students, promoting harmonious group integration (i.e., relatedness support; Leo et al., 2023e). Conversely, teachers who thwart this need create a distant, cold, and unresponsive classroom atmosphere, adopting behaviors of rejection and exclusion toward students, as well as fostering poor relationships among them (i.e., relatedness thwarting; Leo et al., 2023e). In this regard, numerous correlational studies based on SDT (Deci and Ryan, 2000) have found a positive relationship between PE teachers’ relatedness-supportive behaviors and students’ relatedness satisfaction in PE, as well as between PE teachers’ relatedness-thwarting behaviors and students’ relatedness frustration (Vasconcellos et al., 2020). In turn, relatedness satisfaction, understood as the experience of feeling connected, valued, and cared for in one’s social environment, has been positively related to autonomous motivation and affective (e.g., enjoyment), behavioral (e.g., engagement), and cognitive (e.g., academic performance) domains, while relatedness frustration, which refers to the perception of being excluded, rejected, or ignored in interpersonal contexts, has been positively associated with problematic relationships (i.e., affective domain), disruptive behaviors (i.e., behavioral domain) and low academic performance (i.e., cognitive domain; Leo et al., 2023e; Sparks et al., 2017; Vasconcellos et al., 2020).

Therefore, grounded in SDT, the role of teachers in promoting positive socio-emotional classroom climates and shaping students’ interactions is highly significant. However, SDT conceptualizes relatedness as the subjective experience of feeling connected, accepted, and cared for by significant others, and it does not require interaction with the entire peer group for this need to be fulfilled (Deci and Ryan, 2014). That is, a student may satisfy their need for relatedness through a few meaningful relationships, without necessarily feeling integrated into the broader classroom community. This limitation makes it difficult for SDT alone to fully capture group-level dynamics such as class cohesion. Given that Spanish educational policies emphasize active methodologies and peer collaboration, fostering cooperation and positive peer relationships across the classroom is essential to optimize learning. In this context, the concept of class cohesion offers a complementary perspective that focuses on the collective experience of the classroom as a social unit, which has received significantly less attention in educational research (Leo et al., 2023d). Consequently, integrating the CMC allows us to address the theoretical and practical gap left by SDT in capturing the dynamics of peer-to-peer relationships on a class-wide scale.

### Class cohesion from the Conceptual Model of Cohesion

Cohesion was defined by Carron et al. (1998) as “a dynamic process that is reflected in the tendency for a group to stick together and remain united in the pursuit of its instrumental objectives and/or for the satisfaction of member affective needs” (p. 213). In the educational context, class cohesion refers to students’ collaboration in achieving shared academic goals and common social bonds (Leo et al., 2022). This class cohesion can be observed in two main dimensions: task cohesion, which pertains to students’ collaboration in academic activities, and social cohesion, which relates to the strengthening of interpersonal and affective bonds among students (Leo et al., 2022).

In this regard, teachers also play a fundamental role in facilitating class cohesion within the classroom. They can implement strategies to enhance both task and social cohesion through structured activities inside and outside the classroom, fostering collaboration among students. These strategies may include assigning interdependent roles, responsibilities, and tasks, as well as promoting peer communication, peer assessment with formative purposes, and social skills development. Moreover, establishing shared goals, designing challenges that require mutual support, and creating opportunities for students to engage in meaningful interactions, where they can express their interests, concerns, and motivations, help cultivate an integrated and participatory learning environment (Leo et al., 2023d).

Previous research indicates that students who perceive themselves as part of a cohesive group tend to exhibit higher values in key variables related to learning processes, such as students’ autonomous motivation and engagement (Leo et al., 2023a, 2023d, 2023e). Therefore, designing training programs focused on providing PE teachers with relatedness-supportive and class cohesion strategies could foster a warm environment that promotes students’ social interactions and improves learning-related outcomes.

### Previous training programs focused on relatedness support and class cohesion

Previous SDT-based training programs among PE teachers have predominantly focused on how to provide autonomy support to students (Vasconcellos et al., 2020). Additionally, some SDT-based training programs among PE teachers have focused on both autonomy and competence support (Patzak and Zhang, 2025) or have simultaneously addressed all three basic psychological needs—autonomy, competence, and relatedness (Vasconcellos et al., 2020; White et al., 2021). Overall, the results from these studies indicate that teachers who participate in these training programs not only learn how to provide autonomy and competence support in their PE classes but also experience benefits, such as increased need satisfaction at work (Cheon et al., 2014, 2018; Tilga et al., 2021), relatedness satisfaction with their students (Reeve and Cheon, 2021), and greater job satisfaction (Cheon et al., 2014, 2020), among other positive outcomes. Additionally, students taught by trained teachers have demonstrated notable improvements in need satisfaction, need frustration, autonomous motivation and other (mal)adaptive outcomes in PE lessons (Vasconcellos et al., 2020; Reeve and Cheon, 2021). In contrast to these previous works, the present study focuses specifically on relatedness support, based on the premise that meaningful learning can be greatly facilitated through peer interaction within the classroom. Despite the recognized value of peer collaboration for learning and engagement, existing strategies to promote relatedness have typically been implemented alongside other strategies (i.e., autonomy support and competence support; Vasconcellos et al., 2020) and have rarely been applied in isolation (Sparks et al., 2017). As a result, the specific contribution of relatedness support strategies to students’ learning remains largely unexplored (Leo et al., 2023d). To address this gap, the present study integrates two complementary theoretical frameworks (i.e., SDT and CMC). This dual framework allows us to approach relatedness both as an individual psychological experience, in the context of teacher-student and student–student interactions (i.e., SDT), and class cohesion as a shared sense of connection among all classmates as a group (i.e., CMC), in line with the broader educational goal of fostering collaborative and socially rich learning environments.

Despite its theoretical relevance, to our knowledge, only one SDT-based training program in PE has exclusively focused on supporting students’ need for relatedness (Sparks et al., 2017). This cluster-randomized controlled study was conducted in four private Catholic secondary schools, where a three-hour teacher training program was delivered to 10 teachers in a single session. This was followed by two readings provided 1 month later to reinforce the learning. The program was structured into three sequential phases: (a) a theoretical introduction, providing background information on relatedness support; (b) a general presentation, outlining relatedness-supportive strategies using real-life examples and video images and their connection to student motivation; and (c) a last phase in which participants described how they supported relatedness in PE and identified strategies they could realistically implement. After the SDT-based training program, a four-month intervention program took place in PE classes (with two to three sessions per week), integrating relatedness-supportive strategies through different sports activities, basketball, netball, badminton, and Australian football. The results from the study (Sparks et al., 2017) indicated that intervention group teachers were significantly more knowledgeable about relatedness-supportive teaching than control group teachers. Moreover, intervention group students, compared to those in the control group, reported significant improvements in relatedness support from their PE teachers, enjoyment, other-efficacy (i.e., confidence in their teacher’s ability), and peer-focused related-inferred self-efficacy (i.e., perceived peer confidence in one’s abilities). However, no significant changes were observed in self-efficacy, teacher-focused related-inferred self-efficacy (i.e., perceived teacher confidence in one’s abilities), self-determination, and amotivation.

A limitation noted by the authors concerns the structure and duration of the training program. Sparks et al. (2017) suggested that a longer or multi-session intervention might be more suitable to enhance the impact and internalization of the training content by teachers. They also reflect on the need for more engaging and interactive follow-up activities beyond the materials initially provided. Furthermore, all data—except for teachers’ self-reported relatedness-supportive teaching—was gathered via student self-reports. Including additional teacher-report measures, or external observations of teachers’ relatedness-supportive behaviors, could help triangulate the results. Additionally, the study did not include a follow-up post-intervention measure, limiting the possibility to assess the long-term effects for both teachers and students. Finally, sex differences in the study variables were not examined for either students or teachers.

Despite these limitations, the findings reinforce the critical role of teachers in fostering interpersonal relationships among students in PE settings (Gairns et al., 2015). Furthermore, within the CMC, despite an extensive body of literature on intervention studies in sports contexts (Martin et al., 2009; Kwon, 2024), this model has not yet been applied to PE teachers to improve students’ interpersonal relationships (Leo et al., 2023d). Further long-term interventions aimed at effectively fostering students’ sense of relatedness, class cohesion, and learning outcomes in educational contexts are needed.

### Study aims

**Figure 1:**
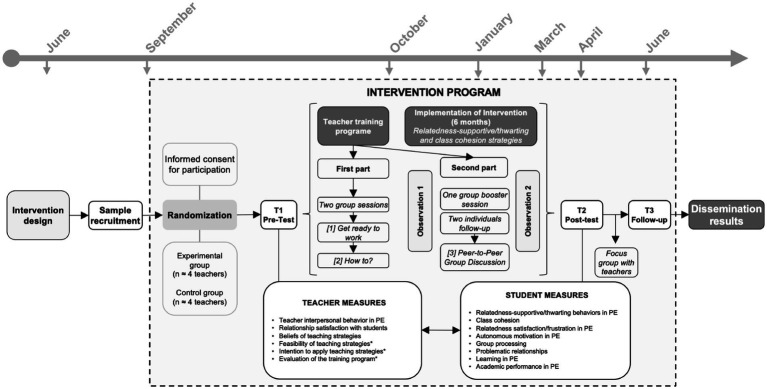
Data collection timeline and overall study design. ^*^Variables measured after the first part of the training program.

Despite the findings outlined above, there is still limited knowledge that has tested the malleability of teacher’ relatedness-supportive/thwarting behaviors and class cohesion and how these teaching practices contribute to students’ interactions and learning processes (Leo et al., 2023d; Vasconcellos et al., 2020; White et al., 2021). Building on this theoretical foundation and the growing interest in relatedness in PE, we developed the TRI-PE Project (*Togetherness, Relatedness, and Interactions in Physical Education*). This project aims to promote relatedness and class cohesion by providing teachers with strategies grounded in SDT and CMC. The acronym reflects the three core pillars of the study: *togetherness*, referring to students’ sense of unity and belonging; *relatedness*, understood as a basic psychological need; and *interactions*, highlighting the role of meaningful social exchanges in the classroom. Therefore, to extend previous knowledge, this mixed-method study describes the TRI-PE Project protocol of a teacher training program based on relatedness-supportive and class cohesion strategies, aimed at improving the instructional practices of PE teachers, as well as students’ interactions and learning-related outcomes (see Figure 1).

To address previous limitations, this study will extend the duration of the teacher training, ensuring that teachers acquire the necessary knowledge and skills to effectively implement relatedness and class cohesion strategies in their classes. To do so, the recommendations proposed by Reeve et al. (2022) for SDT-based training programs will be followed. Additionally, as recommended by Ahmadi et al. (2023), this training program will not only focus on relatedness-supportive behaviors but also on avoiding relatedness-thwarting behaviors, because both have been shown to significantly influence students’ motivational processes and adaptative outcomes (Vasconcellos et al., 2020). Importantly, teachers may learn relatedness supportive strategies while unintentionally maintaining relatedness thwarting behaviors, highlighting the need for awareness and reflection throughout the training. Furthermore, this SDT-based training program will incorporate class cohesion strategies to promote a sense of group belonging, ensuring that all students feel included, which can enhance peer learning and classroom integration.

According to the outcomes, several variables will be selected based on prior evidence identifying them as key motivation- and learning-related constructs (e.g., autonomous motivation, problematic relationships, group processing, learning, or academic performance; Vasconcellos et al., 2020). All study variables will also be examined by sex to determine whether the intervention is equally effective for boys and girls. Finally, a follow-up post-intervention measure will be implemented at the end of the academic year to evaluate the long-term effects of the intervention.

#### Aim 1: effects of teacher training program on students

**Figure 2:**
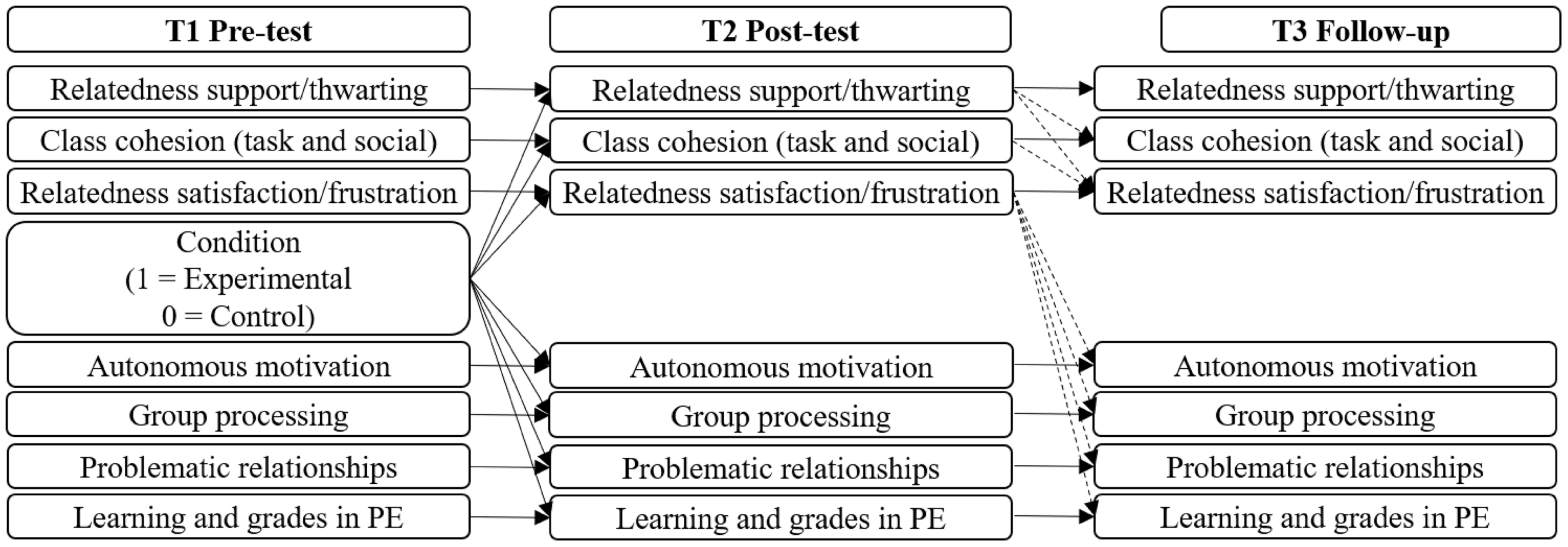
Hypothetical multilevel model of the predictive relationship between the study variables.

Grounded in SDT and CMC, the first aim of this study will be to assess the impact of a teacher training program aimed at enhancing relatedness-supportive behaviors and class cohesion while minimizing relatedness-thwarting behaviors on students’ affective, behavioral, and cognitive outcomes. In line with this aim, three hypotheses will be tested. It is hypothesized that, by Time 2 (T2), experimental group students will perceive improvements in their PE teachers’ relatedness-supportive behaviors (Hypothesis 1), class cohesion, relatedness satisfaction, autonomous motivation, and affective (i.e., problematic relationships), behavioral (i.e., group processing), and cognitive variables (i.e., learning and academic performance; Hypothesis 2) compared to baseline values (Time 1 [T1]) and control group students. Given the lack of previous studies, no specific hypotheses will be formulated regarding the intervention’s long-term effects (Time 3 [T3]) on the study variables. Finally, students’ relatedness satisfaction/frustration and class cohesion, representing an individual-level and a group-level factor, respectively, will serve as mediators in the indirect relationship between the independent variable (i.e., treatment condition) and affective, behavioral, and cognitive outcomes, specifically in autonomous motivation, problematic relationships, group processing, learning, and academic performance (Hypothesis 3, see Figure 2).

Previous correlational studies examining the relationship between students’ perceptions of relatedness support and various outcomes, while accounting for sex differences, have yielded mixed results (Leo et al., 2023b; Leo et al., 2022; Vasconcellos et al., 2020; White et al., 2021). In this regard, although previous studies have included sex as a covariate when analyzing the effects of SDT-based interventions, sex differences are rarely reported for either teachers or students (Vasconcellos et al., 2020). However, systematic reviews and meta-analyses have emphasized the importance of examining school-based motivational intervention effects separately for boys and girls (Vasconcellos et al., 2020). Therefore, it is crucial to account for sex differences when evaluating intervention effects to determine whether they are equally effective for both sexes. Given the inconsistent findings in previous research regarding the study variables, no specific hypotheses will be formulated regarding potential sex differences among students.

#### Aim 2: effects of teacher program on teachers

As for the second objective, most SDT-based training programs in PE have primarily examined their effects on students (Vasconcellos et al., 2020). However, preliminary evidence also suggests that these training programs can be beneficial for teachers as well (Reeve and Cheon, 2021). Thus, using a mixed-method approach, the second aim will be to examine the effects of the training program on teachers’ beliefs, feasibility, and intention to apply relatedness-supportive and class cohesion strategies, use of relatedness-supportive behaviors, and relationship satisfaction with students. In line with this aim, it is hypothesized that, by T2, experimental group teachers will perceive improvement in beliefs, feasibility, and intention to apply relatedness-supportive and class cohesion strategies, use of relatedness-supportive behaviors, and relationship satisfaction with their students, compared to those who do not receive any training (Hypothesis 4). Due to the scarcity of prior research, we will not posit specific hypotheses about the intervention’s long-term (T3) effects on the study variables. If enough male and female teachers participate in the training program, the intervention’s effects on the study variables will also be examined by sex.

#### Aim 3: evaluation of teacher training program

Finally, concerning the third aim, despite the large number of SDT-based training programs in PE (Reeve and Cheon, 2021; Vasconcellos et al., 2020), little attention has been paid to examining the characteristics of the training program itself (Aelterman et al., 2013; Cheon and Reeve, 2015). Examining the acceptance of the training program allows for improving its content before being disseminated in other areas, countries, and contexts. Thus, using a mixed-method approach, the third aim will be to examine the quality of the training program focused on relatedness support and class cohesion strategies. Given that some strategies from previous SDT-training programs will be used (Ahmadi et al., 2023; Reeve et al., 2022), it is hypothesized that experimental group teachers will report positive perceptions of the training program regarding its interaction, innovation, interest, clarity, relevance, and the degree to which they would recommend the training to other educators (Hypothesis 5).

## Method

### Context and design

The TRI-PE project is a randomized controlled trial (RCT) to be conducted in secondary schools within the region of Extremadura. In this region, the academic calendar extends over three terms, from early September to mid-June, with holiday breaks in December–January for Christmas and in March or April for Easter. Specifically, PE is mandatory for all secondary students who attend two coeducational 50-min weekly classes. As illustrated in Figure 1, the intervention will last 10 months, with assessments conducted at three time points: (1) baseline measurement at the end of the first month of the school year (first term, end of September; T1) to ensure that students have had several weeks to a fine-grained picture of the variables under investigation; (2) post-implementation assessment (second term, April; T2); and (3) 2-month follow-up post-implementation at the end of the academic year (third term, June; T3). This study has been approved by the Ethics Committee of University of Extremadura [114/2023] and complies with the principles of the Declaration of Helsinki.

#### Sample calculation

Before conducting this study, a sample size calculation was performed to determine the number of students required for the intervention. The calculation was based on the formula: *n* = (Z) 2 (p (1- p) e2), where *n* represents the number of participants, *Z* is set at 1.96 for a 95% confidence interval, *p* corresponds to the total number of students from 1st year of Secondary Education to 1st year of Upper Secondary Education in the target region (approximately 58,491 students in the 2024/2025 academic year), and *e* represents a 5% margin of error. Considering a potential 10% non-response rate, the minimum sample size required is 385 students.

#### Sample recruitment with inclusion criteria

Between 8–10 secondary PE teachers, assigned to the experimental group and to the control group equally, along a subset of their students, will be anticipated to take part in this study. Given the research team’s limited resources, the maximum number of participating teachers will be set at ten. Randomization will be performed using a digital tool. To minimize the risk of cross-contamination between conditions, teachers from the same high school will not participate. Teachers will be recruited through multiple channels, including social media platforms, telephone calls, and emails. Specifically, information (e.g., flyers and recruitment letters) will be shared via professional networks, PE-related social media groups, and the regional government’s official teacher registry. School principals will also be contacted directly to help disseminate the invitation within their schools.

The registration period will last approximately 2 weeks. Teachers whose participation is not approved by their school leadership will not be included. If more than 10 teachers, or multiple PE teachers from the same school, express interest, a waiting list will be created. Final selection will be based on predefined inclusion criteria and logistical feasibility, including school location, number of participants per school, schedule compatibility, and the research team’s capacity to monitor and support the intervention.

Teachers must meet the following inclusion criteria to participate in this study: (1) be an in-service PE teacher at the same school for the entire academic year; (2) complete the study questionnaires at three different time points; (3) not participate during the entire academic year in other intervention or training programs similar to this study; (4) attend all the training program sessions; (5) allow research team visits for classroom observation; and (6) participate in a focus group at the end of the intervention. Each PE teacher will recruit at least four classroom groups of no fewer than 16 students each, ensuring that at least 80% of the students in each group participate. Eligible students will be those aged 12–18 years from secondary schools. Participation will be completely voluntary and anonymous. School principals and students’ families will also be informed about the study’s aims and characteristics to obtain their informed consent for participation. The 80% participation rate applies only to baseline (T1); teachers not reaching it may be excluded, with replacements drawn from a waiting list. Dropout over time will not lead to exclusion if at least 60% of students complete all data points. The research team will make every effort to retain the full sample and minimize attrition throughout the study. Finally, the inclusion criteria for students’ participation in the intervention program will be: (1) obtaining consent from both parents/legal guardians and students; (2) commitment to completing the questionnaires assessing the study variables three times; and (3) regular participation in PE lessons.

#### Blinding

Experienced research assistants, who are postdoctoral staff and trained university researchers, will conduct all data collection. To preserve blinding, they will not have access to group allocation lists, and school codes will be used during assessment. A separate coordinator will schedule sessions to avoid accidental exposure. Teachers, due to their involvement in training, will be aware of their assignment, but not of the study hypotheses. Students will remain unaware of both the hypotheses and their teachers’ group allocation.

### Teachers’ training program and implementation phase with students

The intervention will consist of two phases for the experimental group: (1) a face-to-face teacher training phase, which includes two group sessions, one group booster session, and two individual follow-up sessions, and (2) an implementation phase, during which teachers will apply the strategies with their students (see Figure 1).

#### Teacher training program

Experimental PE teachers will participate in a training program designed to help them provide relatedness-supportive and class cohesion strategies while avoiding relatedness-thwarting behaviors. The training will be delivered by three experienced researchers in designing and implementing motivational teacher development programs, ensuring a close trainer-to-teacher ratio (~3 trainers per 4–5 teachers) to support implementation quality and fidelity.

##### Face-to-face teacher training phase

The training will be structured into two main parts (see Figure 1) and following the three-phase framework proposed by Reeve et al. (2022): (1) *Get Ready to Work*: theoretical foundations and awareness-building, (2) *How To?*: practical strategy development, and (3) *Peer-to-Peer Group Discussion*: collaborative reflection after implementation. This structure was selected for its evidence-based support in promoting long-term teacher behavior change and alignment with the intervention’s goals.

The first part, which will be conducted in late September, involves two group sessions, each lasting 3 h, that addressed the first two phases (i.e., [1] Get Ready to Work, and [2] How To?). After completing this phase, a round of classroom observations will be conducted to assess the implementation of the proposed strategies. The second part, a booster session, which will be delivered in January, focuses on the third phase (i.e., [3] Peer-to-Peer Group Discussion). This session will be informed by the first observations and teachers’ weekly logs and will be followed by a second round of observations to support teachers’ development and practice integration further. Following this structure, the training sessions will include several activities aimed at enhancing teachers’ theoretical understanding, co-creation of strategies, and practical application, as detailed below.

##### First group session

The first group session will address the initial phases of the training (i.e., [1] Get Ready to Work, and [2] How To?), focusing on strengthening teacher relationships, introducing the study’s aims, and familiarizing teachers with the theoretical frameworks underpinning the intervention (i.e., SDT and CMC). First, teachers will report their prior knowledge and experiences with relatedness-supportive and class cohesion strategies, and then complete the questionnaires on the study variables (T1). To introduce theoretical frameworks, teachers will then reflect individually, using color-coded post-its to sort their relatedness-support and class-cohesion strategies into three groups: yellow for consistently used, blue for occasional use, and pink for desired but not yet applied. Subsequently, teacher motivational behaviors (TMBs) related to relatedness-supportive and relatedness-thwarting (Ahmadi et al., 2023, see Supplementary Table 1) and guidelines for promoting task and social cohesion (Leo et al., 2023d, see Supplementary Table 2) will be presented. Teachers’ identified strategies will then be mapped onto these frameworks to facilitate their integration into practical instruction. The session will conclude with a discussion on the benefits of adopting relatedness-supportive practices and class cohesion strategies, followed by assigning a complementary reading to deepen participants’ theoretical understanding before the second training session (Leo et al., 2023d).

##### Second group session

In the second group session, as part of Phase 2: “How To?,” teachers—drawing on the theoretical frameworks, TMBs, and the assigned reading—will continue to co-create practical strategies that reinforce students’ sense of belonging and class cohesion (i.e., task and social cohesion). In the last part of the sessions, strategies will be evaluated for feasibility, refined by consensus, and integrated into teaching plans for subsequent implementation.

##### Group booster session

Approximately 3 months after the start of the implementation, teachers will participate in a booster session aligned with Phase 3 (i.e., [3] Peer-to-Peer Group Discussion). This timing is designed to allow teachers sufficient opportunity to apply, experiment with, and refine the intervention strategies in real classroom settings. According to Reeve et al. (2022), teachers require a minimum of 1 month of practical experience to integrate new techniques effectively, engaging in processes of experimentation, trial-and-error, reflection, discussion, and collaborative problem-solving. The booster session will address implementation barriers based on teachers’ weekly logs and the first follow-up observation. Teachers will collaboratively explore solutions and engage in simulated PE teaching scenarios (i.e., video recordings of real PE lessons) to reinforce the application of relatedness-supportive and class cohesion strategies.

##### Individual follow-up sessions

In addition to group sessions, two individual follow-up sessions (before and after the booster) will be held between each teacher and a member of the research team. These one-on-one meetings will serve to personalize support, address context-specific issues, and offer tailored feedback based on classroom observations. During each follow-up session, the research team will use a structured protocol to assess the implementation of relatedness-supportive behaviors and class-cohesion strategies. After each observation cycle, an individualized report will be generated for each teacher, providing a detailed analysis of their instructional practices regarding the intervention aims. Specifically, the report will include: (1) an overview of the strategies effectively employed during the two observed lessons; (2) a set of additional relatedness-supportive and class cohesion strategies that could have been implemented to further enhance students’ sense of belonging, peer interactions, and class cohesion; and (3) tailored recommendations for future application and refinement of these strategies. These reports are intended as formative tools to help teachers reflect critically on their practice, identify areas for growth, and adjust instruction in alignment with the intervention framework.

#### PE implementation for experimental group students

The implementation phase will last approximately 6 months, from October to the end of March, as we plan to conduct a post-intervention follow-up measure in the final month of the academic year (i.e., June; see Figure 1). During this period, the co-created strategies will be implemented by the experimental group teachers. Relatedness-supportive and class cohesion strategies learned during the training program will be encouraged for implementation in the different teaching units of their annual teaching plan. Efforts will be made to ensure that teachers implement them as much as possible, in terms of variety, frequency, and intensity, in each PE class. The experimental group teachers will implement a common set of strategies; however, teachers will have the flexibility to adapt these strategies to their specific educational context, considering the characteristics and needs of their students. This process will be conducted under the supervision of a research team member.

#### Control group teachers and students

Teachers in the control group will not initially receive the training program and, consequently, will not intentionally implement any strategies aimed at supporting relatedness and class cohesion with their students. Their participation will only involve completing the questionnaires at the same time points as the experimental group teachers and students (see Figure 1). However, after the final assessment of the study, control group teachers will be invited to receive the same training program, enabling them to implement these strategies in the following academic year. A final report will also be prepared, outlining each teacher’s profile based on the teachers’ self-reports and their students’ perceptions. The training offered to control group teachers after the study will be identical in structure and content but will not involve additional data collection, as it is intended solely to offer equitable access to professional development opportunities.

#### Fidelity of the training program and intervention implementation

Firstly, an external researcher with expertise in SDT-based program design will attend every training session to assess compliance with the prescribed structure and strategies (i.e., congruent style). In addition, he will provide external feedback to the research team members implementing the program.

Secondly, the fidelity of the intervention will be examined in two ways. On the one hand, teachers will submit a weekly checklist via Google Forms, specifying which relatedness-supportive or -thwarting strategies and class-cohesion techniques they have implemented. This will allow teachers and researchers to be aware of the degree of implementation of the various strategies. On the other hand, an observer with experience in identifying TMBs in PE classes will observe two random classes from each PE teacher to assess the degree of implementation of the strategies. For this purpose, three different checklists will be used: (1) the same verification checklist that teachers complete weekly, which includes the co-created strategies developed during the training phase; (2) a checklist based on the TMBs for supporting and thwarting relatedness proposed by Ahmadi et al. (2023; see Supplementary Table 1); and (3) a checklist derived from the guidelines for promoting class cohesion developed by Leo et al. (2023d; see Supplementary Table 2). This multi-instrument approach will provide a more comprehensive assessment of fidelity and ensure the evaluation is aligned with both theoretical constructs and contextualized pedagogical practices.

### Measures

Various assessment instruments will be administered to all study participants at three different time points (September [T1], April [T2], and June [T3]; see Figure 1). During the administration of the students’ questionnaires, at least one research team member will be present in the classroom to address any questions or concerns, and teachers will be absent. Students will be informed that their responses are anonymous, and that neither teachers nor school staff will have access to individual data. The paper-based questionnaire will take approximately 15 min for both students and teachers to complete at each time point.

#### Teachers’ measures

Teachers will self-report their age, sex, teaching experience, type of school (public or private), and school location (rural or urban). It is important to note that, since some teachers’ responses may vary depending on the classroom group, teachers will need to complete the questionnaire with consideration of the specific student groups selected for the study.

##### Teacher interpersonal behavior

To assess teachers’ need-supportive and need-thwarting behaviors, the PE Spanish version of the Interpersonal Behaviors Questionnaire (Burgueño and Medina-Casaubón, 2021) will be adapted to teachers. The scale begins with the stem “In PE classes … “followed by 24 items (four items per factor) that measure autonomy (e.g., “… I encourage them to make their own decisions”), competence (e.g., “… I acknowledge their abilities to achieve their goals”), and relatedness support (e.g., “… I show interest in their activities”), as well as autonomy (e.g., “… I restrict their ability to make decisions”), competence (e.g., “… I question their ability to make progress”), and relatedness thwarting (e.g., “… I do not show interest in them”). It is important to note that although this study primarily focuses on supporting relatedness-supportive behaviors and avoiding relatedness-thwarting behaviors, we will also include autonomy- and competence-supportive/thwarting behaviors to control for their potential effects. Responses will be assessed using a 5-point Likert scale, ranging from 1 (*strongly disagree*) to 5 (*strongly agree*).

##### Relationship satisfaction with students

To assess teachers’ perception of their relationship satisfaction with their students, a single-item measure used in previous studies (Cheon et al., 2020) will be used: “I have a good and satisfying relationship with my students.” Teachers will rate this item on a 10-point Likert Scale, ranging from 1 (*strongly disagree*) to 10 (*strongly agree*).

##### Teacher beliefs

To evaluate teachers’ perceptions of the importance and value of implementing relatedness-supportive and class cohesion strategies during PE classes, the Aelterman et al. (2014) questionnaire will be used. Specifically, this questionnaire has been slightly modified to target relatedness-supportive practices, consisting of four items (e.g., “I believe it is essential for teachers to always promote positive relationships among classmates”). Responses will be assessed using a 5-point Likert scale, ranging from 1 (*strongly disagree*) to 5 (*strongly agree*).

##### Feasibility of teaching strategies

To evaluate teachers’ perceptions of the feasibility of implementing relatedness-supportive and class cohesion strategies, the same set of items used to measure their teaching beliefs (e.g., “It is feasible for teachers to foster positive relationships in the classroom”; Aelterman et al., 2014) will be applied. Responses will be assessed using a 5-point Likert scale, ranging from 1 (*totally unfeasible*) to 5 (*totally feasible*).

##### Intention to apply teaching strategies

To evaluate teachers’ intention of implementing relatedness-supportive and class cohesion strategies, the same four-item scale used to assess their pedagogical beliefs will be employed (e.g., “I will promote positive relationships among classmates”; Aelterman et al., 2014). Responses will be assessed using a 5-point Likert scale, ranging from 1 (*no intention*) to 5 (*definite intention*).

##### Evaluation of the training program

A multi-method approach will be used, combining teacher questionnaires, a focus group with teachers, and a mixed-method assessment by an external researcher. This approach seeks to gather both quantitative and qualitative evidence on the program’s effectiveness and implementation. Firstly, consistent with previous studies (Aelterman et al., 2013), teachers will complete a brief questionnaire assessing various aspects of the training program at the end of the first two group face-to-face sessions. The questionnaire comprises items evaluating the training’s acceptability across six dimension: interaction (i.e., “The training was sufficiently interactive”), innovation (i.e., “The training was innovative”), interest (i.e., “The training was engaging and interesting”), clarity (i.e., “The content was easy to understand”), relevance (i.e., “The training was essential for my learning”), and the degree to which they would recommend the training to other educators (e.g., “I would suggest this training to my colleagues”). Responses will be assessed using a 5-point Likert scale, ranging from 1 (*strongly disagree*) to 5 (*strongly agree*).

Secondly, upon completion of the training program and concurrent with the post-test (T2) administration of both teachers’ and students’ questionnaires, all experimental PE teachers will participate in a focus group. The discussions will address the following key topics: (1) the project’s theoretical underpinnings (i.e., SDT and CMC) and their feasibility within the educational setting; (2) the design and application of strategies aimed at fostering relatedness and class cohesion in PE; (3) the pedagogical approach employed (e.g., use of images, videos, practical examples, formative assessment techniques, and interactive exercises) and teachers’ perceptions of their instructional style; (4) perceived changes in the teachers’ variables assessed via questionnaires; and (5) an overall evaluation of the training program (e.g., innovation, practical relevance, feasibility of motivational strategies, intention to implement them, and overall satisfaction). The focus group session will be led by a principal moderator and an assistant, both with expertise in SDT, CMC, PE, and qualitative methods, with no trainers present to encourage open dialogue. The session will begin with an introduction to the aims and process, followed by a brief overview of the key topics. The assistant will handle logistics, take notes, and manage recordings. Finally, the moderator will summarize the main points and confirm with the teachers if the summary reflects their views or if they wish to add anything. The session will be in a neutral, comfortable setting, lasting approximately 60 min. The session will be recorded and transcribed for analysis.

Third and finally, a mixed-method evaluation will be conducted by an external researcher using the same teacher questionnaire (i.e., interaction, innovation, interest, clarity, relevance, usefulness, and feasibility), and will include external observations of the various sessions. This alignment will enable a more in-depth assessment of the training program’s quality by triangulating teachers’ self-reported perceptions with external observations.

##### Evaluation of the intervention’s effects on students

The focus group will also explore teachers’ perceptions of how the training program and its implementation may have led to changes in students’ outcomes. To this end, specific questions will be included to gain a qualitative perspective on the possible transfer and effects of the training on students’ educational experience.

#### Students’ measures

Students will self-report their age, sex, sociocultural background, and school grade level.

##### Relatedness-supportive/thwarting behaviors

To assess students’ perception of their teacher’s relatedness-supportive and relatedness-thwarting behaviors in PE lessons, the Teacher Interpersonal Style Questionnaire (TISQ; Leo et al., 2023f) will be used. The scale begins with the stem “In PE classes, my PE teacher …” followed by 24 items (four items per factor). In this study, only the relatedness-support factor (e.g., “…always fosters positive relationships among classmates”) and the relatedness-thwarting factor (e.g., “…creates a classroom atmosphere that I do not like”) will be used. Additionally, to control for the effects of the other two basic psychological needs, one item related to autonomy support (e.g., “…takes our opinions into account when planning lessons”) and one item related to competence support (e.g., “…encourages us to trust in our ability to do things well”) will be included. Responses will be collected using a 5-point Likert scale, ranging from 1 (*strongly disagree*) to 5 (*strongly agree*).

##### Class cohesion

To analyze students’ perceptions of class cohesion, the short version of the Class Cohesion Questionnaire (CCQ; Leo et al., 2023c) will be used. This scale consists of six items (three per factor), assessing task cohesion (e.g., “We are united in class during the development of tasks and activities”) and social cohesion (e.g., “Classmates participate in activities outside of class together”). Responses will be collected on a 9-point Likert scale, ranging from 1 (*strongly disagree*) to 9 (*strongly agree*).

##### Relatedness satisfaction

To evaluate students’ relatedness satisfaction in PE lessons, the Spanish version in PE lessons (Moreno-Murcia et al., 2009) of the Exercise Needs Satisfaction Scale (Vlachopoulos and Michailidou, 2006) will be used. The scale starts with the statement “In PE classes.,” followed by four items grouped under a single factor (e.g., “… I feel that I interact with my classmates in a very friendly way”). Responses will be recorded on a 5-point Likert scale, ranging from 1 (*strongly disagree*) to 5 (*strongly agree*).

##### Relatedness frustration

To measure students’ relatedness frustration in PE lessons, the adapted Spanish version of the Psychological Needs Frustration Scale (Bartholomew et al., 2011), validated for the PE context by Trigueros et al. (2020), will be used. This scale begins with the stem phrase: “In PE classes…” and includes four items (e.g., “I feel other people dislike me”). Students will rate each item on a 5-point Likert scale, ranging from 1 (*strongly disagree*) to 5 (*strongly agree*).

##### Autonomous motivation

To assess students’ perceptions of autonomous motivation in PE lessons, the Motivation Questionnaire for Physical Education Classes (CMEF; Sánchez-Oliva et al., 2012) will be used. This questionnaire begins with the phrase “I participate in PE classes…,” followed by eight items evaluating autonomous motivation (e.g., “…because I find it enjoyable and interesting”). Students will respond on a 5-point Likert scale, ranging from 1 (*strongly disagree*) to 5 (*strongly agree*).

##### Group processing

To assess students’ perceptions of group processing, the Cooperative Learning Measurement Questionnaire in Educational Contexts (Fernandez-Rio et al., 2017) will be used. This factor is headed by the stem “In PE classes.,” followed by four items (e.g., “…We engage in group discussions to ensure that everyone understands what is being done”). Responses will be rated on a 5-point Likert scale, from 1 (*strongly disagree*) to 5 (*strongly agree*).

##### Problematic relationships

To assess students’ perceptions of problematic relationships in the classroom, the Problematic Relationships Scale (PRS; Cheon and Jang, 2012) will be used. This one-factor scale consists of four items (e.g., “I feel uncomfortable when interacting with my classmates”). Responses will be recorded using a 5-point Likert scale, ranging from 1 (*strongly disagree*) to 5 (*strongly agree*).

##### Learning in PE

To examine students’ perceived learning, the Perceived Learning Questionnaire (PLQ, Llanos-Muñoz et al., 2025) will be used. The questionnaire begins with the phrase “In PE classes.,” followed by eight items distributed into two factors (four items per factor): acquired learning (e.g., “…I learn things I did not know before”) and functional learning (e.g., “…I learn important things that I can apply in my daily life or the future”). Responses will be measured using a 5-point Likert scale, ranging from 1 (*strongly disagree*) to 5 (*strongly agree*).

##### Academic performance in PE

To measure academic performance in PE lessons, two questions will be used: “What grade did you receive in PE last year/trimester?” and “What grade will you receive in PE this term?.” Following Spanish educational assessment policy guidelines (Spanish Government, 2020), response options will be structured on a five-level scale: 1. Fail, 2. Pass, 3. Good, 4. Very Good, 5. Excellent. These questions have been previously used in educational research (see Guntern et al., 2017; Luo et al., 2023).

### Plan of analysis

A mixed-method analysis will be conducted to evaluate the training program, as well as to assess the impact of its implementation in PE lessons on the study variables for both teachers and students. Regarding the quantitative analysis, first, a descriptive analysis will be conducted to evaluate both teachers’ and the external researcher’s perceptions of the training program, assessing aspects such as its innovation, practical relevance, and the feasibility of the strategies implemented. Second, to assess the impact of the intervention program on the study variables for both teachers and students, the nature of the variables will first be examined through tests of normality, validity, and reliability across the three measurements. Third, the assumptions of independence, linearity, and homogeneity of variance will be verified to ensure the appropriateness of analyses based on the general linear model. Fourth, between-group and within-group differences will be analyzed using repeated-measures multivariate analysis of covariance (MANCOVA). A 3 × 2 (Time × Condition) repeated measures MANCOVA will be performed for both teachers and students, with covariates such as age, sex, teaching experience, type of school, and school location for teachers, and age, sex, sociocultural background, and school grade for students. If parametric assumptions are not met, appropriate non-parametric alternatives such as the Wilcoxon signed-rank test (for within-group comparisons) and the Mann–Whitney U test (for between-group comparisons) will be used. Subsequently, to examine intragender differences of the intervention on study variables, a 3 × 2 × 2 (Time x Condition x Sex) repeated measures MANCOVA will be performed for both teachers and students. To further examine changes in each outcome variable over time (T1, T2, and T3) within each group (experimental vs. control), multiple paired t-tests with Bonferroni correction will be conducted. Effect sizes will be calculated using partial eta squared (*η^2^ₚ*). Effect size thresholds will be interpreted as small (*η^2^ₚ* > 0.01), moderate (*η^2^ₚ* > 0.06), or large (*η^2^ₚ* > 0.14) following Cohen’s criteria. For all analyses, the significance threshold will be set at *p* < 0.05. All statistical analyses will be conducted using IBM SPSS Statistics v.25.0. Finally, a longitudinal structural equation model will be employed through the statistical software Mplus (Muthén and Muthén, 1998-2019) to analyze the predictive relationships between the study variables, allowing for the observation of potential differences at the three specific times (T1, T2, and T3) when data are collected.

Regarding the qualitative analysis, the qualitative data from the focus group will be transcribed and managed using NVivo Version 11.0 to ensure systematic organization. A thematic analysis following the procedures outlined by Braun and Clarke (2019) will be employed. Initially, three researchers will independently examine the transcripts to familiarize themselves with the content. Relevant excerpts reflecting teachers’ views on the training program’s outcomes and its application with students will then be identified. After coding, final themes and subthemes will be refined to accurately capture the core meanings. The analysis will adopt a deductive approach, grounded in SDT and the CMC, given most of the questions align with these theoretical frameworks. Additionally, two researchers will oversee the process, contributing their insights to support consensus in the interpretation of results.

## Discussion

This study protocol describes a training program for in-service secondary PE teachers and their subsequent intervention implementation with students. Based on SDT (Ryan and Deci, 2017) and the CMC (Leo et al., 2023d) theoretical frameworks, the training program is focused on relatedness-supportive and class cohesion strategies while reducing relatedness-thwarting behaviors. The intervention will assess the benefits of the training program for both students and teachers. Additionally, the study will evaluate the quality of the training program from both the teachers’ and an external researcher’s perspective.

This study is expected to contribute to new knowledge in 10 key areas: (1) grounded in SDT and CMC, this will be the first teaching program specifically designed to equip teachers with relatedness-supportive/thwarting and class cohesion strategies within an educational context; (2) the quality of the training program will also be assessed using questionnaires, a focus group involving all PE teachers, and observations by an external researcher; (3) the training program will be implemented not only prior to the intervention phase but also during it through a booster sessions; (4) the training program will also include two individual follow-up sessions, during which constructive feedback will be provided, along with individualized debriefings; (5) promising strategies that have demonstrated effectiveness in previous SDT-based training programs will be employed (e.g., supportive attitude, congruent style, concise theoretical input, co-creation of teaching strategies; Vasconcellos et al., 2020; White et al., 2021); (6) intervention fidelity will be evaluated using an observation instrument aligned with both SDT and CMC frameworks; (7) the TMBs identified by Ahmadi et al. (2023) will be applied during the intervention to determine which specific techniques drive its effects; (8) the effects of the intervention on teacher variables will be evaluated using a mixed-methods approach, including questionnaires and focus group; (9) the differential effects of the intervention on male and female students —and, if feasible, on teachers— will be examined; and (10) a follow-up post-intervention assessment will be conducted to evaluate the long-term impact on the study variables. These 10 key aspects will help future PE researchers and teachers replicate the training program and its implementation in other educational contexts, ensuring its adaptability and feasibility within typical school settings.

In line with these contributions, the effectiveness of the intervention will be examined through a series of research hypotheses. According to Hypothesis 1, students in the experimental group are expected to show greater improvements in their perception of relatedness support and relatedness thwarting from their PE teacher compared to baseline values and the control group students at post-test (T2). Similarly, it is anticipated that experimental group students will show greater improvements in class cohesion, motivational variables (i.e., relatedness satisfaction/frustration and autonomous motivation), and affective (i.e., problematic relationships), behavioral (i.e., group processing), and cognitive variables (i.e., learning and academic performance) compared to baseline values and control group students (Hypothesis 2).

Additionally, it is expected that students’ relatedness satisfaction/frustration and class cohesion will mediate the effect of the program on affective, behavioral, and cognitive outcomes (Hypothesis 3). All these expected outcomes from the first three hypotheses are supported by scientific literature, as correlational evidence suggests that students’ perceptions of relatedness-supportive behaviors are associated with positive outcomes in PE (Leo et al., 2023e; Sparks et al., 2017; Vasconcellos et al., 2020), while relatedness-thwarting behaviors are linked with maladaptive outcomes (Leo et al., 2023e). Similarly, class cohesion (both task and social cohesion) has been positively linked to educational outcomes, such as motivation and classroom engagement (Leo et al., 2023a, 2023f). Although SDT-training programs specifically aimed at for PE teachers that focus on relatedness-need supportive strategies remain scarce, the only identified study in this area has reported some positive post-intervention outcomes, especially among students (Sparks et al., 2017). Thus, by expanding on previous research through the inclusion of relatedness-thwarting behaviors and class cohesion strategies and addressing its limitations, the study expects to achieve the positive outcomes outlined above.

Finally, we expect that teachers participating in the training program will report improvements in their beliefs, perceived feasibility, and intention to apply relatedness-supportive and class cohesion strategies; an increased use of relatedness-supportive behaviors; and greater satisfaction in their relationships with students (Hypothesis 4 and Hypothesis 5). A systematic review by Reeve and Cheon (2021) suggested that, despite the still limited evidence, teachers could also benefit from such interventions in areas like autonomy support, job satisfaction, and their relationships with students.

### Limitations

This intervention faces certain limitations that should be considered. Firstly, although the first part of the training program can be considered brief to ensure that PE teachers effectively integrate these techniques into their PE classes, an excessively long duration could discourage their participation. Nevertheless, a booster session will be scheduled 3 months after the first two sessions of the training program. Secondly, to avoid overburdening students, we will assess their perceptions of teachers’ autonomy- and competence-supportive/thwarting behaviors with a single item to control for its effects in the analysis, which may limit our ability to fully gauge their impact. In contrast, teachers will complete the full set of items for both need-supportive and need-thwarting behaviors to control for these effects in the analysis. Thirdly, while students’ perceptions of class cohesion will be measured using a validated scale (Leo et al., 2023c), we were unable to assess class cohesion support, understood as the specific teacher behaviors aimed at promoting task cohesion (e.g., “The teacher encourages us to help each other during the development of tasks and activities) and social cohesion (e.g., “The teacher encourages us to participate together in activities outside of class.”). Fourthly, to assess the fidelity of the intervention, only two classes will be randomly observed for each experimental group teacher due to a lack of human resources. Finally, although all teachers will receive the same training program, each teacher will need to adapt the strategies to their context, the characteristics of their students, available spaces, material resources, etc. Therefore, the number and intensity of the use of relatedness supportive and class cohesion strategies may vary slightly depending on each group.

## Conclusion

The present study provides a detailed account of the protocol for a training program aimed at in-service PE teachers, grounded in SDT and CMC, to ensure both transparency and replicability. The training program will help PE teachers support relatedness and class cohesion behaviors while minimizing the use of thwarting-relatedness teaching behaviors. Specifically, the program seeks to improve teachers’ beliefs, perceived feasibility, and intention to implement relatedness-supportive and class cohesion strategies; to increase the actual use of relatedness-supportive behaviors; and to raise teachers’ satisfaction in their interactions with students. This, in turn, may lead to an improvement in class cohesion, motivational-related variables and affective, cognitive, and behavioral outcomes in students. If the results are promising, this study could lay the groundwork for the ongoing development of motivational training programs for in-service PE teachers.

## Data Availability

The original contributions presented in the study are included in the article/Supplementary material, further inquiries can be directed to the corresponding author.
