# Correction: Randomized controlled trial protocol on enhancing students' togetherness, relatedness, and interactions for learning in physical education: the TRI-PE project

**DOI:** 10.3389/fpsyg.2025.1704075

**Published:** 2025-11-13

**Authors:** Rubén Llanos-Muñoz, Javier Sevil-Serrano, David Lobo-Triviño, Miguel A. López-Gajardo, Francisco M. Leo

**Affiliations:** 1 Departmento de Didáctica de la Expresión Musical, Plástica y Corporal, Facultad de Formación del Profesorado, Universidad de Extremadura, Cáceres, Spain; 2 Departmento de Didáctica de la Expresión Musical, Plástica y Corporal, Facultad de Ciencias del Deporte, Universidad de Extremadura, Cáceres, Spain

**Keywords:** group dynamics, intervention, need-support, peer interactions, psychological needs, relatedness support

The reference for “Leo et al., 2023f” was erroneously written as “Leo, F. M., Sánchez-Oliva, D., Fernández-Rio, J., López-Gajardo, M. A., and Pulido, J. J. (2023f). Revista Mexicana de Psicología, in press”.

The correct reference appears below:

“Leo, F. M., Sánchez-Oliva, D., Fernández-Rio, J., López-Gajardo, M. A., and Pulido, J. J. (2023). Validation of the teaching interpersonal style questionnaire in physical education. *Rev. Mexic. Psicol*. in press. doi: 10.31219/osf.io/s6e8a

An incorrect **Funding** statement was provided. The previous statement appears below:

“The author(s) declare that financial support was received for the research and/or publication of this article. Financial support provided by the European Regional Development Fund (ERDF), Government of Extremadura (Counsel of Economy and Infrastructure, Research Groups SEJ048 and SEJ059) and Ministry of Science and Innovation (grant number FPU21/04682)”.

The correct **Funding** statement reads:

“The author(s) declare that financial support was received for the research and/or publication of this article. The author(s) declare that the article has been co-financed at 85% by the European Union, European Regional Development Fund, and the Regional Government of Extremadura, Managing Authority, Ministry of Finance (GR24155, Research Groups SEJ048 and SEJ059), and the Ministry of Science, Innovation and Universities (grant number FPU21/04682)”.

Affiliation Departmento de Didáctica de la Expresión Musical, Plástica y Corporal, Facultad de Formación del Profesorado, Universidad de Extremadura, Cáceres, Spain was omitted for author Miguel A. López-Gajardo. This affiliation has now been added for author Miguel A. López-Gajardo.

Author Miguel A. López-Gajardo was erroneously assigned to affiliation Departmento de Didáctica de la Expresión Musical, Plástica y Corporal, Facultad de Ciencias del Deporte, Universidad de Extremadura, Cáceres, Spain. This affiliation has now been removed for author Miguel A. López-Gajardo.

The original version of this article has been updated.

