# Supplementary material for: Randomized controlled trial protocol on enhancing students’ togetherness, relatedness, and interactions for learning in physical education: the TRI-PE project
Source: Front Psychol. 2025 Aug 26;16:1629158. doi: 10.3389/fpsyg.2025.1629158 (PMC12418117; doi:10.3389/fpsyg.2025.1629158)
Supplement: Supplementary file 1 [file Table_1.docx]

**Supplementary Table 1**

*Checklist of Relatedness Need-Supportive and Need-Thwarting Strategies based on Teacher Motivational Behaviors proposed by Ahmadi et al. (2023)*

|  |  | **Yes** | **No** | **Remarks** |
| --- | --- | --- | --- | --- |
| Relatedness support | 1. Show unconditional positive regard |  |  |  |
|  | 2. Ask about students' progress, welfare, and/or feelings |  |  |  |
|  | 3. Expressing affection |  |  |  |
|  | 4. Promote cooperation |  |  |  |
|  | 5. Teacher enthusiasm |  |  |  |
|  | 6. Show understanding of the students’ point of view |  |  |  |
|  | 7. Group students with similar interests |  |  |  |
| Relatedness thwarting | 1. Ignoring students | | | |
|  | 2. Use abusive language (content) | | | |
|  | 3. Provide punishments unfairly | | | |
|  | 4. Yell or use a harsh tone | | | |
|  | 5. Provide rewards unfairly | | | |
|  | 6. Be sarcastic | | | |
|  | 7. Provide conditional positive regard | | | |
|  | 8. Apply fair punishments | | | |

**Supplementary Table 2**

*Checklist of Guidelines for promoting Class Cohesion based on Leo et al. (2023d)*

|  |  | **YES** | **NO** | **Remarks** |
| --- | --- | --- | --- | --- |
| Guidelines to promoting class cohesion | 1. Educational projects that involve the whole class |  |  |  |
|  | 2. Group dynamics |  |  |  |
|  | 3. Collaborative activities to be developed in class |  |  |  |
|  | 4. Rotation of groups and roles |  |  |  |
|  | 5. Moments to share opinions, ideas, and present them |  |  |  |
|  | 6. Encourage ongoing peer support among students |  |  |  |
|  | 7. Group evaluation processes |  |  |  |
